# Isolation and characterization of microsatellite loci for *Rhododendron shanii* (Ericaceae)

**DOI:** 10.1002/aps3.1222

**Published:** 2019-02-21

**Authors:** Tao Pan, Ya‐Li Pei, Kai Zhao, Xin‐Yue Liu, Chen‐Cheng Wang, Bao‐Wei Zhang

**Affiliations:** ^1^ School of Life Sciences Anhui University Hefei Anhui Province People's Republic of China; ^2^ School of Life Sciences Anhui Normal University Wuhu Anhui Province People's Republic of China; ^3^ College of Resources and Environment Anqing Normal University Anhui Province People's Republic of China

**Keywords:** Ericaceae, genetic diversity, microsatellite, polymorphism, *Rhododendron shanii*

## Abstract

**Premise of the Study:**

We developed microsatellite primers for *Rhododendron shanii* (Ericaceae), a narrowly distributed species found in the Dabie Mountains, China, to study the genetic diversity, population structure, and evolutionary history of the species.

**Methods and Results:**

Two terminal sequencing modes of the Illumina HiSeq platform were used to mine simple sequence repeat markers from large‐scale transcriptional groups. In this study, 24 microsatellite loci were screened. The number of alleles ranged from one to 20, and the levels of observed and expected heterozygosity ranged from 0.000 to 1.000 and 0.000 to 0.918, respectively. Most of these primers were successfully amplified in eight congeneric species (*R. annae*,* R. chihsinianum*,* R. decorum*,* R. denudatum*,* R. fortunei*,* R. neriiflorum*,* R. rex*, and *R. simiarum*).

**Conclusions:**

These newly developed microsatellite loci will be useful for studying the genetic diversity and population structure of *R. shanii* and congeneric species.

*Rhododendron* L. (Ericaceae, Ericales) is a genus of 1024 species of evergreen or deciduous woody plants in the heath family that are mainly distributed in Asia and the highlands of the Appalachian Mountains in North America. Most species have bright flowers that bloom from the end of winter to early summer. One member of the genus, *R. shanii* W. P. Fang, is endemic to the southern Dabie Mountains (Zhao et al., 2010). According to the IUCN Red List of Threatened Species (IUCN, 2018), *R. shanii* is classified as Vulnerable (VU) (Zhao et al., 2012). Analysis of the genetic diversity of endangered species is a primary focus of conservation biology because it can provide crucial information for the protection of rare and endangered species. In recent decades, microsatellites (also known as simple sequence repeat [SSR] markers) have been widely used as genetic markers for population genetics, phylogeography, and conservation genetics due to their high abundance, high levels of polymorphism, codominance, and transferability (Moriguchi et al., 2015). SSR markers have previously been developed for several species in *Rhododendron* (e.g., Delmas et al., 2011; Liu et al., 2017). However, as there are thousands of species in *Rhododendron*, these existing primers are not sufficient for population genetic research, particularly in the rare species *R. shanii*. In addition, although inter‐simple sequence repeat (ISSR) markers have been used to examine genetic diversity in *R. shanii*, these studies have not clearly revealed the genetic structure and population demographics of this species (Zhao et al., 2013). Therefore, it is important to develop microsatellite loci for *R. shanii* to facilitate extensive genetic studies of this species.

Here we developed a set of SSR markers to probe the genetic diversity in five wild populations of *R. shanii*, thereby guiding the conservation of this species. In addition, we tested the versatility and polymorphism of these primer sets in eight congeneric phylogenetically related species of *Rhododendron* (i.e., *R. annae* Franch., *R. chihsinianum* Chun & W. P. Fang, *R. decorum* Franch., *R. denudatum* H. Lév., *R. fortunei* Lindl., *R. neriiflorum* Franch., *R. rex* H. Lév., and *R. simiarum* Hance).

## Methods and Results

Total RNA was extracted from dried leaves of multiple *R. shanii* plants (DZJ population; Appendix 1) using an EasyPure Plant RNA Kit following the manufacturer's instructions (TransGen Biotech Inc., Beijing, China). RNA integrity and quality were evaluated by agarose gel electrophoresis and spectrophotometry. A cDNA library was prepared using a TruSeq Stranded Total RNA Sample Prep Kit (Illumina, San Diego, California, USA), and sequencing was performed using the Illumina HiSeq 3000 platform (Illumina) as described by Berkman and Edwards (2012). For quality control of raw data, Trim Galore version 0.4.4 (Babraham Bioinformatics, http://www.bioinformatics.babraham.ac.uk/projects/trim_galore/) was used to dynamically remove those low‐quality fragments with 10% chance of error in the base call from the 3′ ends of the sequencing data. The quality of the pre‐processed data was analyzed using FastQC version 0.11.5 (Andrews, 2010). A total of 78,354 contigs were obtained by de novo assembly using Trinity version 2.3.2 (Grabherr et al., 2011). All of the assembled contigs were searched for microsatellite loci using BatchPrimer 3 (You et al., 2008), with minimum repeat number of six for dinucleotides and five for tri‐, tetra‐, penta‐, and hexanucleotides. Primer sets were designed with Primer3 with default parameters (Untergasser et al., 2012). A total of 25,564 SSRs were identified from 23,264 contigs and used to design 17,251 microsatellite primer sets. Of these, 24 primer sets that amplified di‐ and tetranucleotide repeats with a minimum of five repeats (range 5–26) were selected at random to test for polymorphism. Raw transcriptome data were deposited in the National Center for Biotechnology Information (NCBI) Short Read Archive (SRA; BioProject no. PRJNA451306, BioSample no. SAMN08967869).

One hundred *R. shanii* samples were collected from five populations (Baimajian [BMJ], Duozhijian [DZJ], Shibigou [SBG], Tianhejian [THJ], and Tuojian [TJ]; Appendix 1). The leaves collected from one individual were regarded as a single sample. A modified cetyltrimethylammonium bromide (CTAB) method was used to extract total genomic DNA from 0.20 mg of leaf tissue (Wang et al., 2010). Each PCR amplification reaction contained 1 μL of DNA (100 ng), 0.5 μL of each primer (10 μM) (forward primer fluorescently labeled with FAM, HEX, or TAMRA; Table 1), 7.5 μL of 2× EasyTaq PCR Supermix (TransGen Biotech), and 5.5 μL of sterilized deionized water in a total volume of 15 μL. Amplification was performed on an ABI 2720 Thermal Cycler (Applied Biosystems, Waltham, Massachusetts, USA) under the following conditions: initial denaturation at 95°C for 5 min; followed by 35 cycles of 30 s at 95°C, at an annealing temperature of 53°C for 30 s, and at 72°C for 20 s; and a final extension at 72°C for 10 min. In order to read the SSR data clearly, the PCR products were individually processed on an ABI PRISM 3730 Genetic Analyzer (Applied Biosystems) with a GeneScan 500 Size Standard and analyzed using GeneMarker (version 1.3; SoftGenetics, State College, Pennsylvania, USA). MICRO‐CHECKER (van Oosterhout et al., 2004) was used to detect the presence of null alleles and errors in the microsatellite genotyping. The number of effective alleles (*A*_e_), observed heterozygosity (*H*_o_), and expected heterozygosity (*H*_e_) were calculated using GENETIX version 4.0 (Belkhir et al., 2001). All population genetic parameters were calculated for each population and across all populations of *R. shanii*. Deviations from Hardy–Weinberg equilibrium (HWE) were tested using GENEPOP version 3.4 (Rousset, 2008) using the following parameters: 10,000 dememorization steps, 20 batches, and 5000 iterations per batches. The function of each locus was determined by a BLAST search of the NCBI database.

**Table 1:** Characteristics of the 24 microsatellite loci developed in *Rhododendron shanii*

Locus	Primer sequences (5′–3′)	Repeat motif	Allele size range (bp)	*T*_a_ (°C)	Function [Organism]	*E*‐value	GenBank accession no.
Rho01	F: TAMRA‐TCCGAGTTCTGATATTGAATGTGT	(TTCT)_5_	265–284	55	Unknown	Unknown	MH211009
	R: AAGACCAAAGTTGCCACCGA						
Rho02	F: TAMRA‐CCAATGCTCGATCTTCTGC	(AAAG)_5_	264–288	55	*Phaseolus vulgaris* hypothetical protein [*Phaseolus vulgaris*]	1e‐64	MH211010
	R: TGCCCAGTTCGTTGTCTAGG						
Rho03*	F: FAM‐GAGTCGGATCGTAGGCTTGG	(CTTC)_5_	178–215	54	Proline rich coiled‐coil 2C [*Seriola lalandi dorsalis*]	5e‐07	MH211011
	R: TTGCAGGGTCAGGGAGAAAG						
Rho04*	F: TAMRA‐GGTGGCGAAGTTGGTAATGC	(GTCT)_5_	248–275	55	Uncharacterized LOC104098765 [*Nicotiana tomentosiformis*]	2e‐72	MH211012
	R: ACTGCGCCCAAGGTTGTTAT						
Rho05*	F: FAM‐TCTCTCTCCCTCCCTTCAGC	(TGTC)_5_	145–164	55	*B. verrucosa* Betv III [*Betula pendula*]	2e‐49	MH211013
	R: TCACTTGAGCCAATCCCAGG						
Rho07	F: HEX‐ACAACACCTACCTTGGAGCG	(TGAT)_5_	180–199	54	Hypothetical protein [*Populus trichocarpa*]	1e‐65	MH211014
	R: TCTCCGTTGCCTTTACCGAC						
Rho09*	F: FAM‐TATATGGCTGGGGTCCGTGA	(TTTA)_5_	130–158	55	Unknown	Unknown	MH211015
	R: ATATGGGCGGATTGGGTTGG						
Rho11*	F: TAMRA‐TGTGTTTCTTCGGCCATGGA	(AGAA)_6_	230–254	55	Unknown	Unknown	MH211016
	R: TCTGTTTACTTGGAATATTGGGTTGT						
Rho12	F: HEX‐CTAGACGAGATCCCCACCCA	(AGAC)_6_	226–268	55	60S ribosomal protein L6‐3‐like [*Raphanus sativus*]	6e‐28	MH211017
	R: GTTGCTGAGCGGGTTTCTTG						
Rho14	F: FAM‐TCACCTCCCTCTCACTCCTC	(TC)_26_	102–155	55	Cultivar Brigitta calmodulin‐1 [*Vaccinium corymbosum*]	5e‐48	MH211018
	R: GATCCGCCATTTCGATTGCC						
Rho15	F: FAM‐AATCCCACCTCTCAAACCCT	(TC)_25_	143–176	55	Unknown	Unknown	MH211019
	R: AGGCTACAAAGAAACGGACGA						
Rho16	F: HEX‐CTCCCCATTCACACAACCCA	(TC)_24_	150–210	55	Auxin early response protein AUX/IAA4 [*Camellia sinensis*]	5e‐144	MH211020
	R: ACCCTCATACAACACGGAGC						
Rho17	F: TAMRA‐TCCCTTTCACTAAACCCTACAGA	(CT)_23_	240–274	56	DEAD‐box ATP‐dependent RNA helicase 53 [*Vitis vinifera*]	7e‐122	MH211021
	R: GCCGGAGATTGCATTTGTGG						
Rho18	F: HEX‐CGTCAGGTGCAAAGGGTTTC	(GA)_23_	151–191	54	Uncharacterized LOC17898248 [*Capsella rubella*]	8e‐67	MH211022
	R: TCTCTTTCTCTTTCCACAACACC						
Rho20	F: FAM‐GAGGGAGATCTCTGTCGGGT	(GA)_21_	96–125	55	Mannosyl‐oligosaccharide 1,2‐alpha‐mannosidase [*Vitis vinifera*]	0.0	MH211023
	R: CTTTGCTTCGGAGTCCTCGT						
Rho21	F: HEX‐GATTGAAGTTCGGCCCAACG	(CT)_21_	160–210	54	Uncharacterized LOC100854951 [*Vitis vinifera*]	1e‐68	MH211024
	R: ACTCTCTCCATCCAAACGACC						
Rho22	F: HEX‐GAGAAGCGGCAGTTGAGAGT	(AG)_21_	201–229	56	Unknown	Unknown	MH211025
	R: TCATCTTCACACACGGCACC						
Rho23	F: FAM‐TCACCTCCCTCTCACTCCTC	(TC)_21_	98–146	55	Cultivar Brigitta calmodulin‐1 [*Vaccinium corymbosum*]	5e‐48	MH211026
	R: GATCCGCCATTTCGATTGCC						
Rho27	F: HEX‐AACACGAACGGCAAAGAACG	(GA)_20_	172–210	55	Sphingoid long‐chain bases kinase 2 [*Cynara cardunculus*]	0.0	MH211027
	R: AGGGACCACAATGAACCTTACA						
Rho29	F: FAM‐TCACCCCACCCATCTCTCAA	(AG)_17_	82–110	55	Transcript variant X1 [*Quercus suber*]	0.0	MH211028
	R: GAAGCACACCCAGTACCCAT						
Rho30	F: FAM‐ TGGCTCTCCTCTTCATTTATTAGAA	(CT)_16_	85–115	56	Unknown [*Diplarche multiflora*]	7e‐17	MH211029
	R: TCATCTTCACACACGGCACC						
Rho31	F: FAM‐CAGGAGATGAGAGACAGCCG	(TC)_16_	106–140	55	Unknown	Unknown	MH211030
	R: AATCACTGCTCCCAACCTCC						
Rho32	F: HEX‐ACAACAACTGGACCCTGCTT	(CT)_16_	126–165	55	Unknown	Unknown	MH211031
	R: AGATGATTGATGGGATGAAGATGA						
Rho33	F: HEX‐TCTCTCCTCTCCATCGATCGT	(GA)_23_	185–235	54	Uncharacterized LOC108988035 [*Juglans regia*]	3e‐39	MH211032
	R: CCGTTCTGGTGTTGCTGTTG						

Note

*T*_a_ = annealing temperature.

* Indicates significant deviation from Hardy–Weinberg equilibrium after Bonferroni correction for the total population (*P* < 0.01).

In this study, we screened 24 microsatellite loci, revealing a total of 291 alleles. Considering all populations, five loci were found to deviate from HWE (*P* < 0.01) in *R. shanii* (Table 1). The nuclear genetic diversity varied between populations, with *A*_e_ ranging from 5.83 (SBG) to 7.54 (THJ), *H*_o_ from 0.650 (SBG) to 0.718 (DZJ), and *H*_e_ from 0.591 (SBG) to 0.685 (THJ). Overall, SBG showed the lowest genetic diversity of the five populations (Table 2). The deviation from HWE was likely related to sample size, substructuring of the samples, or intra‐population inbreeding. Across all five populations of *R. shanii* using the 24 loci, the number of alleles ranged from one to 20, *H*_o_ ranged from 0.000 to 1.000, and *H*_e_ ranged from 0.000 to 0.918 (Table 2). Putative functions were identified for 17 microsatellite loci through BLAST searches (Table 1). We next tested these 24 primer sets for cross‐amplification in *R. annae*,* R. chihsinianum*,* R. decorum*,* R. denudatum*,* R. fortunei*,* R. neriiflorum*,* R. rex*, and *R. simiarum* (Appendix 1). Most of the primers also amplified products in these eight species and no null alleles were found (Table 3).

**Table 2:** Genetic diversity of the 24 newly developed microsatellites in five populations of *Rhododendron shanii*.^a^

Locus	BMJ (*n* = 20)			DZJ (*n* = 20)			SBG (*n* = 20)			THJ (*n* = 20)			TJ (*n* = 20)			Total		
	*A*	*H*_o_	*H*_e_	*A*	*H*_o_	*H*_e_	*A*	*H*_o_	*H*_e_	*A*	*H*_o_	*H*_e_	*A*	*H*_o_	*H*_e_	*A*	*H*_o_	*H*_e_
Rho1	4	0.970*	0.759	4	1.000*	0.616	4	0.885*	0.607	4	0.500*	0.523	4	0.722*	0.627	4	0.800	0.698
Rho2	5	0.515*	0.520	6	0.667*	0.731	5	0.192*	0.251	2	0.467	0.398	3	0.444	0.387	6	0.450	0.481
Rho3	7	0.788	0.812	8	0.697	0.808	6	0.500	0.596	6	0.500	0.785	6	0.583	0.746	8	0.630	0.784
Rho4	6	0.364	0.632	3	0.455	0.363	6	0.346	0.569	5	0.367	0.490	5	0.629	0.684	8	0.400	0.596
Rho5	3	0.152*	0.455	5	0.424*	0.642	2	0.000*	0.145	1	0.000	0.000	2	0.000*	0.407	5	0.061	0.407
Rho7	8	0.606*	0.753	5	0.667*	0.683	4	0.154	0.389	4	0.321	0.611	7	0.361	0.585	10	0.343	0.637
Rho9	2	0.333	0.282	1	0.000	0.000	1	0.000	0.000	3	0.393	0.495	2	0.083	0.081	3	0.163	0.206
Rho11	5	0.182	0.394	2	0.424	0.339	3	0.577	0.446	6	0.393*	0.720	4	0.233	0.445	6	0.344	0.461
Rho12	4	0.212*	0.276	4	0.697*	0.592	2	0.038	0.038	2	0.333	0.325	2	0.278	0.243	4	0.300	0.298
Rho14	10	0.939	0.870	12	1.000	0.897	12	0.923	0.900	13	0.929	0.890	12	0.917	0.868	16	0.939	0.909
Rho15	13	0.969	0.902	12	1.000	0.863	10	0.846	0.827	12	0.828	0.910	12	0.943	0.904	15	0.928	0.901
Rho16	12	0.788	0.858	10	0.656	0.874	9	0.731	0.749	13	0.967	0.845	20	0.972	0.910	20	0.778	0.879
Rho17	12	0.848	0.834	9	1.000	0.837	6	0.654	0.659	13	0.821	0.845	13	0.941	0.853	16	0.842	0.860
Rho18	13	0.844	0.855	9	0.750	0.864	10	0.808	0.869	15	0.963	0.892	14	0.742	0.875	19	0.832	0.885
Rho20	8	0.879	0.798	7	0.970	0.657	7	1.000*	0.716	9	0.931	0.835	9	0.972	0.784	13	0.940	0.778
Rho21	12	0.879	0.809	8	1.000*	0.836	8	0.731	0.741	11	0.929	0.839	10	0.806	0.776	14	0.867	0.844
Rho22	7	0.938	0.697	7	0.938	0.654	6	0.962	0.644	6	0.967	0.703	6	0.970	0.684	9	0.948	0.680
Rho23	10	0.879	0.877	16	0.970	0.918	12	0.885	0.893	14	0.900*	0.901	11	0.833	0.848	16	0.897	0.902
Rho27	8	0.871	0.775	5	0.688	0.761	10	0.731	0.807	11	0.900	0.815	13	0.917	0.846	19	0.776	0.820
Rho29	11	1.000	0.869	5	0.909	0.702	7	0.885	0.729	9	0.867	0.841	9	0.861	0.831	11	0.920	0.814
Rho30	6	0.909	0.638	8	0.758	0.590	4	0.962	0.575	6	0.828	0.606	5	0.833	0.590	10	0.870	0.605
Rho31	6	1.000	0.622	6	0.939	0.683	4	1.000*	0.667	6	0.967	0.754	6	0.917	0.697	10	0.940	0.689
Rho32	13	1.000	0.888	7	0.727*	0.790	9	0.800	0.805	14	0.828	0.851	13	0.972	0.864	16	0.848	0.889
Rho33	12	0.970	0.886	6	0.879	0.776	6	0.962	0.790	12	0.931	0.897	12	0.912	0.851	12	0.918	0.863
Total loci	7.21±0.61	0.707±0.058	0.674±0.040	6.00±0.51	0.718±0.057	0.671±0.042	5.83±0.55	0.650±0.070	0.591±0.051	7.54±0.80	0.712±0.057	0.685±0.044	7.21±0.79	0.700±0.066	0.674±0.044	6.76±0.30	0.697±0.027	0.659±0.020

Note

*A* = number of alleles; *H*_e_ = expected heterozygosity; *H*_o_ = observed heterozygosity; *n* = number of individuals.

a Voucher and locality information are provided in Appendix 1.

* Indicates significant deviation from Hardy–Weinberg equilibrium after Bonferroni correction for each population (*P* < 0.01).

**Table 3:** Cross‐amplification results for the 24 microsatellite loci developed for *Rhododendron shanii* in eight species of *Rhododendron*, as indicated by allele size ranges.^a^^,^ b

Locus	*R. decorum*	*R. chihsinianum*	*R. simiarum*	*R. denudatum*	*R. neriiflorum*	*R. fortunei*	*R. annae*	*R. rex*
Rho1	268–270	268–270	268–280	268–280	266–268	264–268	268	270
Rho2	266–274	266–270	270–276	274–276	276–286	266–270	266–276	270–274
Rho3	185–189	189–203	183–197	189–203	211–213	197–199	187–199	197–199
Rho4	261–263	267–273	257–259	257–259	257–259	257–261	257–259	253–257
Rho5	152–156	152–156	156	156–162	152–162	152–156	152	148–152
Rho7	181–189	181–189	189–197	181–189	189–197	189–197	189–197	189–193
Rho9	—	131	—	—	—	131	—	—
Rho11	235	231–235	231–235	243–247	243–247	243–247	231–235	235–239
Rho12	240–264	254	236	230–234	230	250	230–252	230
Rho14	132–138	126–128	116–124	128–130	112–118	116–126	116–132	120–130
Rho15	150–154	156–158	146–158	144–158	160–162	154	146–154	150–164
Rho16	161–199	177–185	177–203	175–185	151–183	159–163	185–195	175–179
Rho17	251–259	251–163	251–255	247–259	249–261	245–257	247–257	249–257
Rho18	162	176–178	152	158	160	178	160–188	152–158
Rho20	104–108	100–102	98–100	100–102	98–102	100–110	98–100	100–102
Rho21	180	180	194	162–178	178–192	180	180	180
Rho22	202–204	220–222	206	208–218	208–218	206–208	—	208–210
Rho23	132–138	126–128	116–124	126–130	106–112	120–126	98–116	98–130
Rho27	177–179	179–193	191–195	173–177	183–193	177–201	177–181	181–207
Rho29	97–107	93–101	93–101	97–101	85–91	105–107	91–97	89–97
Rho30	87–97	99–101	87–89	85–89	—	85–97	87–97	87–97
Rho31	114–136	124–136	118–136	112	108–118	136	136	—
Rho32	146	146	150–154	146–158	146	128–146	146	152–156
Rho33	209–231	201–211	201–233	187–211	213–215	205–211	193–209	195

Note

— = unsuccessful amplification.

a *N* = 6 for all species.

b Voucher and locality information are provided in Appendix 1.

## Conclusions

We identified 25,564 SSRs from 23,264 contigs and designed 17,251 microsatellite primers. The 24 novel microsatellite markers identified in this study are valuable tools that will be useful for investigating population structure, gene flow levels, and mating systems, as well as for conservation genetic studies of *R. shanii*. These microsatellite primers could also be used to genotype congeneric species (*R. annae*,* R. chihsinianum*,* R. decorum*,* R. denudatum*,* R. fortunei*,* R. neriiflorum*,* R. rex*, and *R. simiarum*).

## Data Availability

Raw transcriptome data were deposited in the National Center for Biotechnology Information (NCBI) Short Read Archive (BioProject no. PRJNA451306, BioSample no. SAMN08967869). Sequence information for the developed primers has been deposited to NCBI; GenBank accession numbers are provided in Table 1.

## Appendix 1 Voucher information for the *Rhododendron* species used in this study.

**Table:**

Species	Population	Collection locality	Geographic coordinates	*n*	Voucher ID^a^
*R. shanii*	BMJ	Baimajian, Anhui, China	31°7′N, 116°11′E	20	AHD‐dz‐bmj‐201703
	DZJ	Duozhijian, Anhui, China	30°59′N, 116° 7′E	20	AHD‐dz‐dzj‐201703^b^
	SBG	Shibigou, Anhui, China	30°58′N, 116° 5′E	20	AHD‐dz‐sbg‐201703
	THJ	Tianhejian, Anhui, China	31°4′N, 116°11′E	20	AHD‐dz‐thj‐201703
	TJ	Tuojian, Anhui, China	30°55′N, 116° 5′E	20	AHD‐dz‐tj‐201703
*R. annae* Franch.		Lushan Botanical Garden, Jiangxi, China	29°51′N, 115°59′E	6	AHD‐ty‐201709
*R. chihsinianum* Chun & W. P. Fang		Lushan Botanical Garden, Jiangxi, China	29°51′N, 115°59′E	6	AHD‐ht‐201709
*R. decorum* Franch.		Lushan Botanical Garden, Jiangxi, China	29°51′N, 115°59′E	6	AHD‐dbh‐201709
*R. denudatum* H. Lév.		Lushan Botanical Garden, Jiangxi, China	29°51′N, 115°59′E	6	AHD‐zy‐201709
*R. fortunei* Lindl.		Lushan Botanical Garden, Jiangxi, China	29°51′N, 115°59′E	6	AHD‐yj‐201709
*R. neriiflorum* Franch.		Lushan Botanical Garden, Jiangxi, China	29°51′N, 115°59′E	6	AHD‐hh‐201709
*R. rex* H. Lév.		Lushan Botanical Garden, Jiangxi, China	29°51′N, 115°59′E	6	AHD‐dw‐201709
*R. simiarum* Hance		Lushan Botanical Garden, Jiangxi, China	29°51′N, 115°59′E	6	AHD‐htdj‐201709

Note

*n* = number of individuals.

a All voucher specimens were collected by Kai Zhao and deposited at Anhui University (AHU), Heifei, Anhui, China.

b Voucher used for RNA extraction.
